# Gene expression profiling of human whole blood samples with the Illumina WG-DASL assay

**DOI:** 10.1186/1471-2164-12-412

**Published:** 2011-08-15

**Authors:** Mary E Winn, Marian Shaw, Craig April, Brandy Klotzle, Jian-Bing Fan, Sarah S Murray, Nicholas J Schork

**Affiliations:** 1 Graduate Program in Biomedical Sciences, Department of Medicine, University of California at San Diego, La Jolla, CA 92093, USA; 2 Scripps Genomic Medicine and Scripps Translational Science Institute, The Scripps Research Institute, La Jolla, CA 92037, USA; 3 Scripps Health, La Jolla, CA 92037, USA; 4 Department of Molecular and Experimental Medicine, The Scripps Research Institute, La Jolla, CA 92037, USA; 5 Illumina Inc., San Diego, CA 92121, USA

## Abstract

**Background:**

Microarray-based gene expression analysis of peripheral whole blood is a common strategy in the development of clinically relevant biomarker panels for a variety of human diseases. However, the results of such an analysis are often plagued by decreased sensitivity and reliability due to the effects of relatively high levels of globin mRNA in whole blood. Globin reduction assays have been shown to overcome such effects, but they require large amounts of total RNA and may induce distinct gene expression profiles. The Illumina whole genome DASL assay can detect gene expression levels using partially degraded RNA samples and has the potential to detect rare transcripts present in highly heterogeneous whole blood samples without the need for globin reduction. We assessed the utility of the whole genome DASL assay in an analysis of peripheral whole blood gene expression profiles.

**Results:**

We find that gene expression detection is significantly increased with the use of whole genome DASL compared to the standard IVT-based direct hybridization. Additionally, globin-probe negative whole genome DASL did not exhibit significant improvements over globin-probe positive whole genome DASL. Globin reduction further increases the detection sensitivity and reliability of both whole genome DASL and IVT-based direct hybridization with little effect on raw intensity correlations. Raw intensity correlations between total RNA and globin reduced RNA were 0.955 for IVT-based direct hybridization and 0.979 for whole genome DASL.

**Conclusions:**

Overall, the detection sensitivity of the whole genome DASL assay is higher than the IVT-based direct hybridization assay, with or without globin reduction, and should be considered in conjunction with globin reduction methods for future blood-based gene expression studies.

## Background

Peripheral whole blood is an attractive source of mRNA for the identification, examination, and development of disease biomarkers via microarray-based gene expression [1]. In fact, many studies have explored the utility of gene expression patterns in whole blood for the purposes of classifying or predicting clinical conditions [2-4]. However, the sensitivity and specificity of microarray assays using peripheral whole blood are reduced due to the relatively high proportion of globin mRNA present in total RNA, which obscures the detection of transcripts expressed at low levels in whole blood [5,6]. While globin reduction assays have been shown to overcome these effects when used in conjunction with Affymetrix microarrays [7] and the standard Illumina direct hybridization assay [8,9], globin reduction assays require large amounts of total RNA [7], fail to completely eliminate globin transcripts [7], and may induce distinct gene expression profiles [10]. Consequently, methods of developing blood-based gene expression biomarker panels that do not involve globin reduction are needed. Developing a microarray-based gene expression assay that does not rely on globin reduction or other methods of sample fractionation, such as the isolation of PBMCs or other cell types from the blood, should reduce sample variability introduced by sample handling and preparation. This will result in a more accurate reflection of the transcriptome at the time of blood draw, and will reduce time and cost.

There are ways to eliminate the need for globin reduction including 1.) the removal of globin probes from the microarray; and 2.) the elimination of globin transcript amplification. Originally developed for the profiling of partially degraded and fixed RNA samples, the highly sensitive and reproducible Illumina cDNA-mediated annealing, selection, extension and ligation (DASL) assay [5,11] uses random priming and a modifiable oligo pool for cDNA synthesis. Random priming in conjunction with PCR amplification may allow for the increased detection of low abundance transcripts. In addition, removing globin-specific oligos from the DASL Assay Oligo Pool (DAP) should decrease noise associated with the high abundance of globin mRNA transcripts and potentially eliminate the necessity of globin reduction. Currently, the DAP is available with and without globin-specific oligos. In order to assess the need for globin reduction with the Illumina DASL assay, we compared microarray gene expression profiles of peripheral blood total RNA and globin-reduced RNA amplified via in vitro transcription (IVT)-based direct hybridization, DASL with globin-specific oligos, and DASL without globin-specific oligos.

## Methods Summary

**Figure 1:**
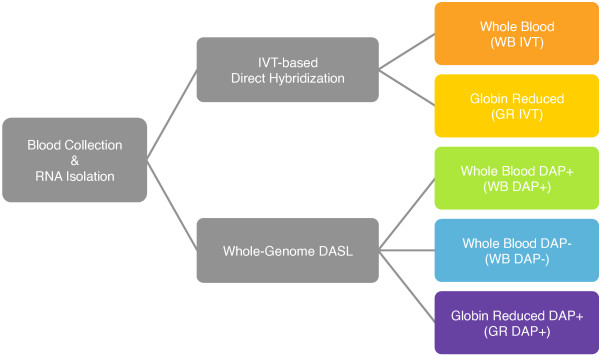
**Flow diagram of study design**. A PAXGene blood tube was collected from 8 individuals then frozen and stored for later processing. RNA was isolated and microarray targets prepared by one of five different methods: IVT-based direct hybridization with total RNA (WB IVT), IVT-based direct hybridization with globin-reduced RNA (GR IVT), whole-genome DAP+ DASL with total RNA (WB DAP+), whole-genome DAP- DASL with total RNA (WB DAP-), and whole-genome DAP+ DASL with globin-reduced RNA (GR DAP+).

Peripheral whole blood samples were collected from eight human donors in PAXGene blood RNA tubes. RNA was isolated after freezing and storage and then prepared for gene expression analysis using the Illumina Human-Ref8 v3.0 Beadchip. Alpha and beta globin were reduced from a portion of the total RNA using the GLOBINclear assay (Ambion, Austin, TX, USA). Two methods of microarray target preparation were examined: Illumina IVT-based direct hybridization (IVT) and Illumina Whole-Genome DASL (WG-DASL) (Figure 1). The differences between IVT and WG-DASL are outlined in Table 1. Two DASL Assay Oligo pools (DAP) were utilized for DASL target preparation: the DASL Assay Oligo Pool with globin probes (DAP +) and the DASL Asssay Oligo Pool without globin probes (DAP-). Comparisons involving the number of genes whose expression levels were detected and the actual levels of expression of the genes were made across the different platforms. A more complete description of the methods is provided in the Methods section.

**Table 1:** Summary of IVT and WG-DASL Methods

	IVT	WG-DASL
**Target Preparation** **Protocol Name**	*In Vitro *Transcription	cDNA-mediated annealing, selection, extension and ligation
**Total RNA Input** **Amount**	50-100 ng	10-200 ng
**Priming Method**	Reverse Transcription off polyA tail	Poly(T) and random priming with biotinylated nonamers
**Amplification**	*In Vitro *Transcription (Linear)	PCR (Exponential)
**Hybridization**	Illumina BeadChip	

## Results

### Comparison between IVT and WG-DASL with and without globin reduction

**Figure 2:**
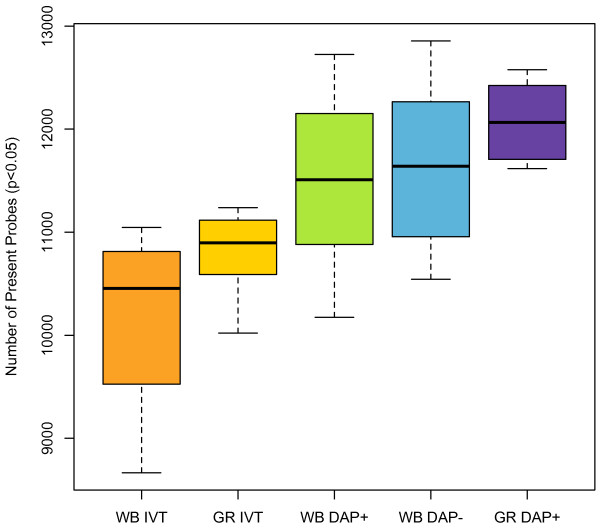
**Box plots of present calls**. The number of detected probes (detection p-value < 0.05) per target preparation method are shown. The boxes represent the lower quartile through the upper quartile, while the whiskers extend to 1.5 times the interquartile range. A bold line denotes the median. WB IVT and GR IVT (n = 8). WB DASL+, WB DASL-, and GR DAP+ (n = 16).

**Figure 3:**
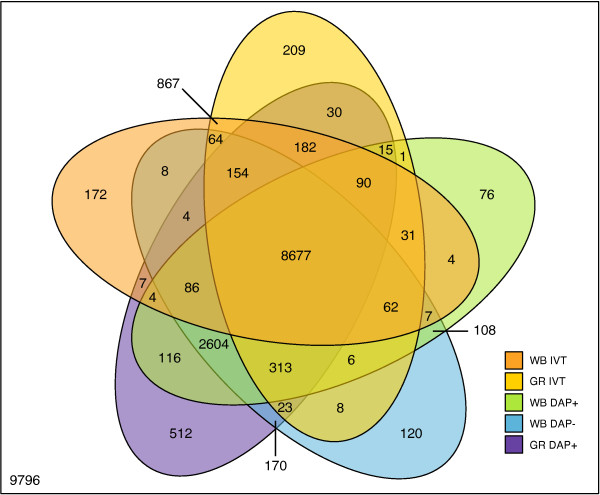
**Overlap of detected probes**. Probes detected as present across all eight samples per target preparation method are compared. WB IVT: IVT-based direct hybridization with total RNA, GR IVT: IVT-based direct hybridization with globin-reduced RNA, WB DAP+: whole-genome DAP+ DASL with total RNA, WB DAP-: whole-genome DAP- DASL with total RNA, and GR DAP+: whole-genome DAP+ DASL with globin-reduced RNA.

Following target amplification as outlined in Figure 1, samples were hybridized with the Illumina Human-Ref8 v 3.0 following the manufacturer's instructions. Each target preparation method was assessed for performance by the number of probes detected as present (Detection p-value < 0.05) (Figure 2). Probes are generally detected as present if the probe intensity is significantly increased in comparison to the array background intensity. As noted, high levels of background due to the presence of globin transcripts in whole blood are known to decrease the number of significantly detected probes. The WG-DASL target preparation method significantly improved detection sensitivity compared to IVT (p-value = 2.13 × 10^-9 ^from an analysis of variance (ANOVA)). Globin reduction decreased probe detection variability with both IVT and WG-DASL target preparation methods. The removal of globin probes from the DASL assay oligo pool (DAP-) resulted in a moderate increase in the number of probes detected but had no significant affect on detection variability (p-value = 0.680, ANOVA) as compared to the DAP+ target preparation method. Overall, 8677 probes were detected across all samples by the five target preparation methods (Figure 3), but only 867 probes were detected by IVT alone. 2604 probes were detected by WG-DASL alone.

**Figure 4:**
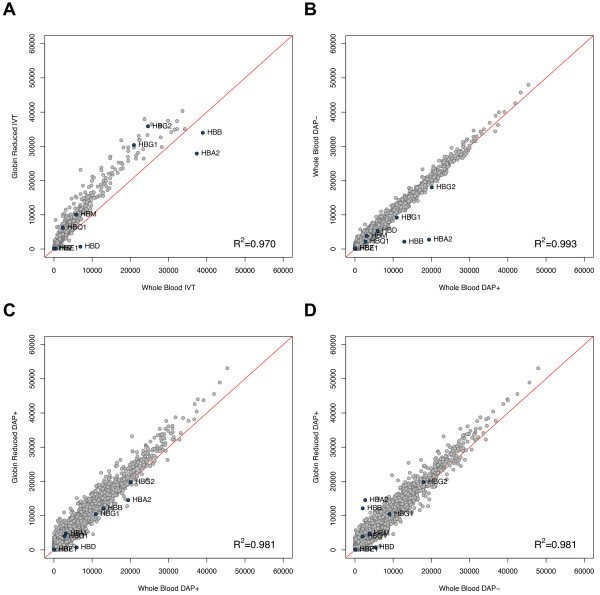
**Raw intensity scatter plots**. Raw intensities for all probes (n = 24526) were compared for (A) whole blood RNA and globin reduced RNA with IVT, (B) whole blood RNA with DAP+ and whole blood RNA with DAP-, (C) whole blood RNA and globin reduced RNA with DAP+, and (D) whole blood RNA with DAP- and globin reduced RNA with DAP-. Correlations for sample 1 are depicted. Average correlations for paired WB IVT versus GR IVT, WB DAP+ versus WB DAP-, WB DAP+ versus GR DAP+, and WB DAP- versus GR DAP- samples are 0.955, 0.992, 0.976, and 0.979, respectively. All 8 hemoglobin genes assayed on Illumina BeadChip Human-Ref v3.0 are labelled: HBA2, HBB, HBD, HBE1, HBG1, HBG2, HBM, HBQ, and HBZ. GLOBINclear specifically targets only HBA2 and HBB for reduction.

NanoDrop Spectrophotometer 260/280 ratios were moderately decreased following globin reduction with an average ratio equal to 2.06 prior and 1.97 post globin reduction (Table 2). However, raw intensity correlations indicate that whole and globin-reduced blood yield similar expression profiles with both IVT and DASL DAP+ assays. Overall raw intensity values increased in globin reduced samples (Figure 4A, C-D) despite the failure of GLOBINclear to completely eliminate the two most abundant globin transcripts, hemoglobin alpha (HBA2) and hemoglobin beta (HBB). The removal of globin probes from the DASL Assay Oligo Pool (DAP-) (Figure 4B) had little effect on gene expression profiles compared to DAP+ (R^2 ^= 0.993) despite the near complete elimination of HBA2 and HBB.

**Table 2:** RNA quality as assessed by 260/280 ratio

Sample ID	Before Amplification	Amplified	1st Globin Reduction	2nd Globin Reduction	Pooled Globin Reduction	Diluted to 20 ng/ul	Average
**C00023 (tRNA)**	2.03	2.01				2.07	2.04
**C00023 (GC RNA)**	1.98	1.99	2.02	1.89	2.00	1.94	1.97
**C00027 (tRNA)**	2.06	2.01				2.06	2.04
**C00027 (GC RNA)**	1.98	2.01	2.02	1.95	1.94	1.89	1.97
**C00169 (tRNA)**	2.04	2.12				1.89	2.02
**C00169 (GC RNA)**	1.96	1.95	1.88	1.90	2.02	1.84	1.93
**C00179 (tRNA)**	2.04	1.99				2.12	2.05
**C00179 (GC RNA)**	1.95	1.98	1.90	2.01	2.01	2.02	1.98
**C00275 (tRNA)**	2.03	1.95				2.14	2.04
**C00275 (GC RNA)**	1.99	1.99	1.87	1.91	1.93	2.01	1.95
**C00304 (tRNA)**	2.05	1.99				2.25	2.10
**C00304 (GC RNA)**	1.98	2.00	1.89	2.11	1.91	1.96	1.98
**C00311 (tRNA)**	2.04	2.10				2.12	2.09
**C00311 (GC RNA)**	2.02	2.00	2.04	1.93	1.97	2.01	2.00
**C00342 (tRNA)**	2.01	2.07				2.21	2.10
**C00342 (GC RNA)**	2.03	2.03	1.97	1.99	1.93	1.97	1.99

RNA quality was assessed before and after globin reduction as well as before and after amplification. tRNA: total RNA; GC RNA: GLOBINclear treated RNA or globin reduced RNA.

### Expression patterns maintained across target preparation methods

**Figure 5:**
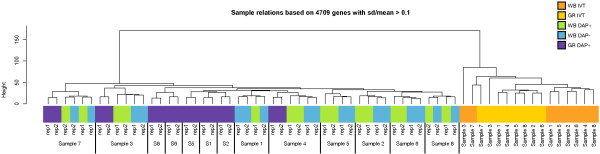
**Sample relations as assessed by unsupervised hierarchical clustering**. Dendrogram reflecting the clustering of the individual samples and the different sample preparation methods. The dendrogram was constructed using hierarchical clustering methods as implemented in the Bioconductor *lumi *package.

IVT target amplification is approximately linear while WG-DASL is approximately exponential, making it difficult to compare expression intensities directly. Thus, it was important in our analyses that the sample-to-sample relations are maintained among each target preparation method. Despite the differences in target amplification, sample relations were preserved across the five target preparation methods as shown by unsupervised hierarchical clustering (Figure 5). For example, with both IVT and WG-DASL, expression profiles for Sample 3 and Sample 7 exhibited the greatest differences from the other six samples, while for the IVT or WG-DASL whole blood RNA clustered separately from globin reduced RNA.

## Discussion

The analysis of whole blood, microarray-based gene expression profiles is often hindered by low sensitivity and high variability due to high levels of globin mRNA transcripts. These issues have been addressed by the development of globin-reduction methods, which specifically target and remove globin transcripts prior to array hybridization. However, studies have shown that globin reduction, like other methods of sample fractionation, may alter expression profiles [10], require large amounts of sample input, increase sample variability [7], and lead to increased costs. Thus, the ability to assay whole blood without sample fractionation or globin reduction may result in improved gene expression profile quality and decrease cost.

Here we describe the utility of a highly sensitive, whole-genome assay in the assessment of whole blood gene expression. Our results suggest that gene expression detection sensitivity is significantly increased with the whole-genome cDNA-mediated annealing, selection, extension and ligation (WG-DASL) assay as compared to IVT-based direct hybridization (IVT). The increased detection sensitivity of WG-DASL may be due to, 1.) random priming allowing for cDNA synthesis along the length of mRNA transcripts, or 2.) the ability to produce larger amounts of cDNA with PCR amplification. Regardless, attempts to further improve detection sensitivity and decrease expression variability through the selective removal of globin probes from the DASL assay oligo pool (DAP-) did not exhibit any large improvements over globin-probe positive DASL (DAP+). Our study also confirms the positive effect of globin reduction on microarray quality when used in conjunction with the Illumina BeadChip and standard IVT-based hybridization [9], while showing that the positive effect of globin reduction extends to WG-DASL as well. However, as shown by unsupervised hierarchical clustering analysis, globin reduction appears to mildly influence gene expression profiles produced by both IVT and WG-DASL assays. Whether this is due to the induction of a globin reduction-specific profile [10], reduced RNA quality due to globin reduction, or the result of decreased noise is unknown, and should be taken into consideration while planning blood-based gene expression experiments.

## Conclusions

Overall, our results suggest that the detection sensitivity of the WG-DASL assay is higher than the IVT-based direct hybridization assay, with or without globin reduction, and should be considered in conjunction with globin reduction methods for future blood-based gene expression studies. However, further investigation into the ability of the WG-DASL assay to distinguish between disease populations using whole blood is needed, as our study was not designed to address such issues.

## Methods

### Blood collection and RNA isolation

For each sample, 2.5 ml whole blood was collected in a PAXgene Blood RNA collection tube (Qiagen, Valencia, CA, USA) and stored frozen at -80°C prior to RNA isolation. RNA isolation was performed using the PAXGene Blood RNA Isolation System (Qiagen, Valencia, CA, USA). RNA quantity and quality were assessed by NanoDrop^® ^Spectrophotometer (Thermo Scientific, Wilmington, DE, USA) before and after globin reduction as well as before and after RNA amplification. For the 8 samples isolated, the total RNA yield ranged from 5.8 - 13.8 ug (average 7.9 ug +/- 1.0 ug), while A260/A280 ratios revealed all samples appeared to be of sufficient quality for microarray analysis (1.93 - 2.10) (Table 2), despite a moderate decrease in quality following globin reduction.

### Globin Reduction

Alpha and beta globin mRNA were reduced from a portion of the total RNA samples using the GLOBINclear™ Human kit (Ambion, Austin, TX, USA) according to the manufacturer's instructions with the recommended start quantity of 2 μg of total RNA. Each sample was processed twice then globin-reduced RNA pooled prior to RNA amplification and hybridization.

### RNA amplification and hybridization

Whole blood total RNA and globin-reduced samples were assayed at both Scripps Genomic Medicine (La Jolla, CA, USA) and Illumina (San Diego, CA, USA) for IVT and DASL-based labelling, hybridization, and scanning, respectively (Table 1). Briefly, the WG-DASL method utilizes biotinylated random nonamer and oligo (dT) primers to convert 10-200 ng input RNA to cDNA. The biotinylated cDNA is then immobilized to a streptavidin-coated solid support and annealed to a pool of gene-specific oligonucleotides (DAP) for extension and ligation followed by PCR amplification with a biotinylated and a fluorophore-labeled universal primer. Finally, the single-stranded PCR products are eluted and hybridized to an Illumina BeadChip. For this study, 250 ng and 100 ng input RNA were utilized for IVT and DASL, respectively.

Gene expression analysis was performed on all whole blood RNA and globin-reduced samples using Human-Ref8 v3.0 Beadchips (Illumina, San Diego, CA, USA) containing 24,526 probes. All arrays were scanned with the Illumina BeadArray Reader and read into Illumina GenomeStudio^® ^software (version 1.1.1). Individual samples were assayed once for all IVT analyses and twice for all DASL analyses. Given the limited amount of mRNA, replicates were only performed for the DASL assay due to its relative novelty as compared to the IVT assay. All replicates were highly correlated (average R^2 ^= .9925). All raw data is available on the NCBI Gene Expression Omnibus (http://www.ncbi.nlm.nih.gov/geo, [GSE 28064]).

### Microarray data analysis

Raw intensities values were exported from GenomeStudio^® ^software (version 1.1.1) for data processing and analysis in R (http://www.R-project.org) and Bioconductor (http://www.bioconductor.org) [12]. Data quality and sample relations were assessed using the Bioconductor *lumi *package [13]. Probes with a Detection p-value less than 0.05 were considered present. Analysis of Variance (ANOVA) was used to assess the consistency of present/absent calls across the different sample preparation methods. Correlation coefficients were calculated from the raw intensity levels to assess the similarity of expression profiles.

## Abbreviations

cDNA: complementary deoxyribonucleic acid; DAP+: DASL Assay Oligo Pool with globin probes; DAP-: DASL Assay Oligo Pool without globin probes; DASL: cDNA-mediated annealing, selection, extension and ligation; GR: RNA following globin reduction by GLOBINclear; HBA2: hemoglobin, alpha 2; HBB: hemoglobin, beta; HBD: hemoglobin, delta; HBE1: hemoglobin, epsilon; HBG1: hemoglobin, gamma A; HBG2: hemoglobin, gamma G; HBM: hemoglobin, mu; HBQ: hemoglobin, theta 1; HBZ: hemoglobin, zeta; IVT: in vitro-transcription; mRNA: messenger RNA; WB: total RNA from peripheral whole blood; WG: whole genome.

## Authors' contributions

MEW participated in the design of the study, performed all data analysis, and drafted the manuscript. MS carried out the IVT-based microarray assays. CA and BK carried out the DASL-based microarray assays. JF and SSM participated in the design of the study. NJS conceived of and participated in the design and coordination of the study and helped draft the manuscript. All authors read and approved the final manuscript.
